# Casino Ownership and Health-Related Community Resources Among Native American Tribes in California

**DOI:** 10.5888/pcd16.180252

**Published:** 2019-02-07

**Authors:** Vanessa M. Oddo, Lina Pinero Walkinshaw, Jessica C. Jones-Smith

**Affiliations:** 1 University of Washington School of Public Health, Department of Health Services, Seattle, Washington; 2 University of Washington School of Public Health, Center for Public Health Nutrition, Seattle, Washington; 3 University of Washington School of Public Health, Department of Epidemiology, Seattle, Washington

## Abstract

**Introduction:**

Casinos are significantly associated with improved health among some Native Americans living on tribal lands. An increase in health-related community resources related to tribal ownership of casinos may be one mechanism through which the health of Native Americans is improved. However, no studies have quantitatively assessed whether casinos are associated with having more community resources.

**Methods:**

To investigate the association between casino ownership and health-related community resources among Native Americans, we surveyed 81 of California’s 110 federally recognized tribes about casino ownership and health-related community resources during 2015 and 2016. We created a total health-related community resources score (maximum of 50 points) by grouping resources into 5 subdomains (community infrastructure, health care and education, social determinants, recreational infrastructure, and recreation programs), which we scored for a maximum of 10 points each and then summed. Casino ownership was our independent variable. We used adjusted linear regression models to test the association between casino ownership and health-related community resources.

**Results:**

Half (49%) of the tribes surveyed owned a casino. Compared with tribes without casinos, tribes with casinos had higher total health-related community resource scores (β = 5.09; 90% confidence interval [CI], 1.17–9.01). Casino-owning tribes had more resources related to community infrastructure (β = 1.81; 90% CI, 0.81–2.80), social determinants of health (β = 1.45; 90% CI, 0.24–2.67) and recreational infrastructure (β = 1.08; 90% CI, 0.24–1.92) compared with tribes without casinos.

**Conclusions:**

Casino ownership is significantly associated with health-related community resources. Future research should assess whether community resources mediate the relationship between economic resources and health among Native Americans.

**Summary**

**What is already known on this topic?**

Casinos have increased economic resources among some Native Americans living on tribal lands, and casinos are associated with improved health.

**What is added by this report?**

This study tests a key hypothesized mechanism through which tribally owned casinos may improve Native Americans’ health. We found that casino ownership was associated with having more health-related community resources, particularly those related to community and physical activity–related infrastructure and social determinants of health.

**What are the implications for public health practice?**

Findings suggest that Native American tribes are successfully investing casino revenue into community resources, which could have implications for improving tribal public health.

## Introduction

Native American–owned casinos were legalized by the US federal government with the Indian Gaming Regulatory Act of 1988. This Act stipulates that profits from Native American–owned casinos are required to be spent on tribal governance — providing for the welfare of the tribe and tribal members, promoting tribal economic development, and funding of local government agencies — or donated to charitable organizations (1). A key goal of Native American gaming is to promote economic development among Native American tribes. Although there can be unintended consequences of opening a casino (eg, higher crime rates) (2), several studies show that economic indicators have improved to a greater degree for tribes with gaming facilities compared with tribes who do not own gaming facilities (3,4).

Additionally, casinos have been significantly associated with improved health outcomes among Native Americans. Among Native American adults, living in a community with a casino has been associated with greater decreases in mortality, obesity, diabetes, smoking, and binge drinking, compared with living in a community without a casino (2,5). Among Native Americans in California, time-series data suggest that living in a community with a Native American–owned casino was significantly associated with decreased childhood obesity (4) and newborn large-for-gestational age (6). Longitudinal findings in North Carolina indicate that the opening of a casino in the community and subsequent improvements in individual income among Native American families was significantly associated with decreased risk of some behavioral issues in childhood (7) and of substance abuse in young adulthood (8).

Increases in family income and health-related community resources related to casinos are hypothesized mechanisms through which tribal casino ownership may improve Native Americans’ health. Many studies indicate that populations with higher incomes and levels of community resources have better health outcomes compared with populations with lower incomes and community resources (9–21). Prior reports and case studies have suggested that tribes often distribute a portion of the profits to tribal members (called per capita payments) and invest profits in community resources that may affect health (22–24). Casinos have been significantly associated with improved per capita income, percentage of the population working but still poor, and the percentage of the population in poverty (2,4,22). However, to our knowledge, no studies have quantitatively assessed whether casinos are associated with having more community resources.

The primary objective of this study was to investigate the relationship between casino ownership and health-related community resources for 81 tribes in California. We hypothesized that owning a casino would be associated with having more health-related community resources, such as physical activity–related infrastructure, health clinics, and resources related to social determinants of health.

## Methods

California has the largest Native American population in the United States, as reported by the 2010 Census. In California, there were 110 federally recognized tribes as of 2016 (25), and 2017 data indicate that 72 tribes now own a casino (also referred to as gaming tribes) (26). In California, a Revenue Sharing Trust Fund (RSTF) was established to redistribute revenues from larger gaming operations to smaller gaming operations and nongaming tribes. Tribes then decide how to invest the profits generated from either their casino or the RSTF. In addition to casinos, other common tribally owned businesses include hotels, gas stations, convenience stores, tourist attractions, and smoke shops.

### Data source and collection

From March 2015 through June 2016, tribal council members and community leaders of California’s federally recognized tribes participated in a survey about health-related resources available in their communities. We developed the survey questions on the basis of qualitative interviews with tribes in California (23) and a review of the literature.

We obtained contact information for 102 tribes, 5 of which were “landless tribes” (ie, at the time of 2010 census, they did not have designated tribal lands). Data were collected via several modes, including mail (n = 17), telephone (n = 11), online (n = 46), or in person (n = 20). First, we contacted 102 tribal leaders and provided them with the option to complete the survey on paper and return by mail or complete it online. We contacted tribal leaders with survey reminders 2.4 times on average. Additionally, 20 surveys were collected in person among participants.

We obtained completed surveys from 94 individuals and 81 unique tribes in California. This response represents 74% of the tribes in California (25) and a response rate of 79%. One tribal survey was excluded from the analyses because the tribe name was not provided. For tribes for whom we received responses from more than 1 tribe member (n = 12 duplicate responses), we selected 1 response per tribe based on the respondent’s seniority. If the respondents had the same level of seniority, then we selected the most complete response. If neither seniority nor completeness were discriminatory, we then selected a response at random. Survey eligibility criteria included self-identification as a “tribal community member qualified to answer questions about health resources available to the tribe.” The survey took 15 minutes to complete. All survey participants received a $50 VISA gift card. Institutional review board review determined that this was not human subjects research.

### Dependent variables

Our primary outcome was the total number of health-related community resources available within each tribe. We created a score to capture the number of health-related community resources by first grouping resources into 5 subdomains: 1) community infrastructure, 2) health care and education, 3) social determinants, 4) recreational infrastructure, and 5) recreation programs. The community infrastructure subdomain included 5 resources: presence of sidewalks, parks, playgrounds, community gardens, and community centers. The health care and education subdomain included 8 resources: tribal health clinics, health insurance, chronic disease prevention programs, healthy living campaigns, wellness programs, nutrition courses and resources, weight loss programs, and health fairs. The social determinants subdomain included 5 resources: housing assistance, college scholarships, promoting higher education, promoting GED programs, and after school programs. Recreational infrastructure comprised 8 resources: community baseball or softball fields, basketball courts, swimming pools, running paths, walking/hiking trails, tribal-owned gyms, gym availability in the community, and sports scholarships. Finally, the recreation program subdomain included 4 resources: fitness classes, school-based sports leagues, community-based sports leagues, and sports tournaments.

We decided that we had no reason to weight any of these subdomains more heavily than the others. Therefore, each subdomain had a maximum of 10 points (range, 0–10), regardless of the number of resources in each subdomain so that, in the total score, each subdomain had equal weight. For example, each resource in the social determinants subdomain was worth 2 points, because the social determinants subdomain comprised 5 resources; whereas each resource within the health domain was worth 1.25 points, because 8 resources made up this domain. To create the total resource score, we summed each of the subdomain scores, for a maximum score of 50 (range, 0–50).

### Independent variables

Casino ownership, a binary self-reported variable, was the primary independent variable. On the basis of literature documenting that casinos generate revenue (2,4,26) and the option for tribes to invest that revenue into the welfare of the tribe or tribal members (as one of 5 ways profits can be spent [1]), we hypothesized that owning a casino would be associated with tribes having higher levels of health-related community resources.

### Covariates

We identified a minimally sufficient set of confounders using a directed acyclic graph. Our confounders included total population living on tribal land and the urbanicity of the tribal land; we controlled for these characteristics in all models. Data on the total population living on tribal lands was obtained from the 2010 Census. Urbanicity was defined according to 2013 Rural–Urban Continuum Codes (RUCCs). RUCCs range from 1 (metro areas) to 9 (completely rural areas). For landless tribes, we imputed total population and urbanicity by using the mean of each variable from the entire sample.

Several studies with quasi-experimental designs confirm that casinos are significantly associated with improved economic resources. In these cross-sectional data, community-level economic development was hypothesized to be a mediator of the casino–community resources association and therefore was not controlled for in these analyses.

The gray literature has documented that operating a casino is significantly associated with having other tribal business enterprises (26). Tribal ownership of other business enterprises may be a proxy for larger, more profitable casinos, or a downstream effect of casino revenue. Therefore, business enterprise ownership was also hypothesized to be a mediator of the casino–community resources association and was not included as a confounder. However, because we think owning other businesses can also result in increased health-related community resources, in secondary analyses, we explored whether tribal ownership of other business enterprises was associated with higher levels of health-related community resources, when controlling for casino ownership. Tribal businesses queried included gas stations, hotels, tourist attractions, convenience stores, and others. Tribal business ownership was defined as a count of all business enterprises that tribes reported (range, 0–4).

### Statistical analyses

In our primary analyses, we used adjusted multivariable linear regression models to test the association between casino ownership and total health-related community resources. We also tested to see if casino ownership was significantly associated with each of the 5 subdomains by using separate adjusted multivariable linear regression models.

In secondary analyses we wanted to estimate the association between additional business enterprise ownership and community resources to ensure that the association was not being driven by casinos, since other businesses could be directly related to the casino or be a proxy for a large casino. To do so, we tested whether other tribally owned businesses were also significantly associated with community resources, when controlling for casino ownership status.

Residual-versus-fitted plots were visually inspected for the final models. We found no indication of problems with functional form; however, in some models, errors looked slightly heteroscedastic. Therefore, we used robust standard errors, which relax the assumption of homoscedastic error terms. *P* was set at < .10, and analyses were performed using Stata 15.1 (StataCorp LP).

## Results

Survey respondents included tribal chairmen and vice chairmen (28%), tribal administrators (21%), council members (17%), community leaders (21%), and treasurers/secretaries (12%) (Table 1). Half (49%) of the tribes owned a casino. Most tribes (63%) provided per-capita payments. Tribes had a median population of 144 (interquartile range [IQR], 56–365) and a median RUCC of 4 (IQR, 1–6).

**Table 1:** Characteristics of Survey Respondents and Tribes (N = 81), Study on the Relationship Between Casino Ownership and Health-Related Community Resources Among Native American Tribes in California, 2015–2016

Characteristic	Value
**Individual respondent tribal role, no. (%)**	
Chairman	17 (21)
Vice chairman	6 (7)
Council member/representative	14 (17)
Treasurer/secretary	10 (12)
Administrator	17 (21)
Community leader/other	17 (21)
**Tribal casino ownership, no. (%)**	
Yes	40 (49)
No	41 (51)
**Tribal per capita payments, no. (%)**	
None	30 (37)
<$1,000/y	11 (14)
≥$1,000/y	40 (49)
**Reservation median rural–urban continuum code^a^, interquartile range**	4 (1–6)
**Reservation median total population^b^, interquartile range**	144 (56–365)

a Defined based on the United States Department of Agriculture’s 2013 Rural–Urban Continuum Codes.

b The total population living on tribal lands from the 2010 Census data.

Most (75%) tribes with casinos reported owning other business enterprises as compared with 34% of tribes without casinos (Table 2). The mean health-related community resources score was higher among tribes with casinos (total score = 31) compared with tribes without casinos (total score = 24).

**Table 2:** Tribe Characteristics, by Casino Ownership, Study on the Relationship Between Casino Ownership and Health-Related Community Resources Among Native American Tribes in California, 2015–2016

Characteristic	Tribe Owns a Casino (n = 40)	Tribe Does Not Own a Casino (n = 41)
**Tribal enterprise ownership, no. (%)**		
No other tribal business enterprises	10 (25)	27 (66)
Tribe owns other business enterprises	30 (75)	14 (34)
**Health-related community resources, mean (standard deviation)**		
Total score	31 (12)	24 (10)
Subdomain scores		
Community infrastructure	5.70 (2.95)	3.61 (2.50)
Health care and education	6.09 (3.34)	5.85 (3.39)
Social determinants of health	8.25 (3.14)	6.54 (2.93)
Recreational infrastructure	4.16 (2.75)	2.57 (2.23)
Recreation programs	6.75 (3.06)	5.37 (3.52)

In our primary models, tribal ownership of a casino was associated with having a higher total health-related community resource score (β = 5.09; 90% confidence interval [CI], 1.17–9.01) (Table 3). We found that owning a casino (versus not) was significantly associated with having more resources in the community infrastructure (β = 1.81; 90% CI, 0.81–2.80) and the recreational infrastructure (β = 1.08; 90% CI, 0.24–1.92) subdomains. Owning a casino (versus not) was also significantly associated with tribes having more resources related to social determinants of health (β = 1.45; 90% CI, 0.24–2.67). Owning a casino (versus not) was not significantly associated with having health care and education (β = 0.02; 90% CI, −1.29 to 1.33) or recreational programming (β = 0.73; 90% CI, −0.44 to 1.90) resources.

**Table 3:** Linear Regression for the Relationship Between Casino Ownership and Health-Related Community Resources Among Native American Tribes in California (N = 81), 2015–2016^a^

Item	Casino Ownership	
	β (90% Confidence Interval)	*P* Value
Total resources	5.09 (1.17 to 9.01)	.03
Community infrastructure	1.81 (0.81 to 2.80)	.003
Health care and education	0.02 (−1.29 to 1.33)	.98
Social determinants of health	1.45 (0.24 to 2.67)	.05
Recreational infrastructure	1.08 (0.24 to 1.92)	.04
Recreational programs	0.73 (−0.44 to 1.90)	.30

a All linear regression models were adjusted for total population and urbanicity.

Owning additional business enterprises was significantly associated with a higher total health-related community resource score, when controlling for casino ownership (β = 2.55; 90% CI, 1.16–3.95) (Table 4). Similarly, owning additional business enterprises was significantly associated with having more resources related to community infrastructure (β = 0.63; 90% CI, 0.23–1.03), social determinants of health (β = 0.68, 90% CI, 0.22–1.15), and recreational infrastructure (β = 0.64; 90% CI, 0.35–0.94), when controlling for whether or not a tribe owned a casino. Owning additional business enterprises was not significantly associated with having resources related to health care and education (β = 0.20; 90% CI, −0.32 to 0.72) or recreational programming (β = 0.40; 90% CI, −0.05 to 0.84).

**Table 4:** Linear Regression for the Relationship Between Tribal Enterprise Ownership and Health-Related Community Resources Among Native American Tribes in California (N = 81), Controlling for Casino Ownership, 2015–2016^a^

Item	Tribal Enterprise Ownership	
	β (90% Confidence Interval)	*P* Value
Total resources	2.55 (1.16 to 3.95)	.003
Community infrastructure	0.63 (0.23 to 1.03)	.01
Health care and education	0.20 (−0.32 to 0.72)	.52
Social determinants of health	0.68 (0.22 to 1.15)	.02
Recreational infrastructure	0.64 (0.35 to 0.94)	.001
Recreational programs	0.40 (−0.05 to 0.84)	.14

a All linear regression models were adjusted for total population, urbanicity, and casino ownership. Tribal enterprise ownership included gas stations, hotels, tourist attractions, convenience stores, and other and was defined as a count of all business enterprises that tribes reported (range, 0–4).

## Discussion

We found that tribes who own casinos tended to have more health-related resources overall compared with tribes who do not own casinos, and they had more resources related to community infrastructure, recreational infrastructure, and social determinants of health. Our findings also suggested that tribal ownership of additional business enterprises is significantly associated with having more health-related community-level resources.

Our study had limitations. First, the data were cross-sectional, so we do not know whether casino ownership preceded community health-related resources, and we cannot infer that any associations are causal. Similarly, unmeasured confounders could have influenced both the development of a casino and the development of health-related community resources. However, we controlled for community-level urbanicity and tribe size, both of which could influence casino ownership and community resources. Third, our survey may not have captured all data on available health-related community resources; however, few respondents wrote in an “other” response that was not written into the survey. Fourth, this survey was not validated, and the results may be specific to the use of this particular scale.

Nevertheless, owning a casino was significantly associated with more resources overall and in particular, resources related to community and recreational infrastructure, such as community centers, running paths/trails, and gyms. This finding is consistent with a related qualitative study, which found that tribal members perceived that per-capita payments and improved cash flow, stemming from casino profits, contributed to improved community and physical activity-related infrastructure (23). Participants highlighted the construction of new sports facilities after casinos were built in their communities (23). 

Our findings are also consistent with the broader literature indicating that lower-income communities tend to have limited access to trails, parks, and recreational facilities (9,10,12–14). Estabrooks and colleagues found that parks, sports facilities, gyms, community centers, and trails were less prevalent in lower-income (versus higher-income) communities (9). Similarly, lower neighborhood-level median household income and a higher prevalence of racial/ethnic minority populations are significantly associated with fewer physical fitness facilities and sports club memberships (12). In simulation models, Powell and colleagues showed that moving from a community with a median household income of $25,000 to $75,000 would increase the likelihood of having physical fitness facilities and sports club memberships by 17% and 38%, respectively (12). 

Availability of and proximity to recreation facilities and the walkability of the community environment is significantly associated with higher levels of physical activity among adults (27) and children (10). In turn, higher physical activity levels are significantly associated with lower obesity prevalence among adults (28) and children (10,29). Drawing on several bodies of literature and our findings, we speculate that casino revenue may improve the weight-related health of Native Americans through increases in community and physical activity-related infrastructure.

Our findings provide support for the idea that owning a casino was significantly associated with tribes having additional resources related to social determinants of health (eg, promoting higher education). Kodish et al similarly found that tribal members in California perceived that casino revenue was used to create college scholarship funds for tribal members (23). Paired with previous findings that indicate that casinos have improved income levels for Native American communities (2,4,22), our findings are also consistent with literature suggesting that higher-income communities tend to offer more education-related resources. Schools in higher-poverty districts tend to have lower school-level expenditures (15) and fewer after-school programs (16). Relatedly, some evidence suggests that lower-income areas have fewer early childhood education and care services (17–20), whereas higher-income neighborhoods tend to have more enriching after school activities (30). If casinos do cause additional community-level resources for education and/or housing, these could also be a plausible mechanism of the casino–health association.

In our secondary models, ownership of additional tribal businesses was significantly associated with more community resources overall and the domains of community infrastructure, recreational infrastructure, and social determinants of health. The fact that owning additional tribal businesses was significantly associated with more community resources, even when controlling for casino ownership, suggests that additional community resources related to general infrastructure, recreational infrastructure, and social determinants of health could be a result of revenue stemming from business enterprises, regardless of whether a tribe owns a casino. In other words, additional community resources could stem from casino revenue, tribal business revenue, or both.

Literature has documented that casinos have increased economic resources among some Native Americans living on tribal lands and that casinos are significantly associated with improved health. We found that casino ownership was significantly associated with health-related community resources, particularly those related to community and physical activity–related infrastructure and social determinants of health. Future research should assess the extent to which community resources mediate the relationship between economic resources and health among Native Americans using causal research methods.
